# Attitudes and behavioral response toward key tobacco control measures from the FCTC among Chinese urban residents

**DOI:** 10.1186/1471-2458-7-248

**Published:** 2007-09-18

**Authors:** Tingzhong Yang, Yanwei Wu, Abu Saleh M Abdullah, Di Dai, Fuzhong Li, Junqing Wu, Haiqing Xiang

**Affiliations:** 1 Center for Tobacco Control Research, Zhejiang University School of Medicine, Hangzhou, 310058, China; 2 Institute of Health Education, Chinese Center for Disease Control and Prevention; 3 Department of International Health, Boston University School of Public Health, Boston, Massachusetts, USA; 4 Oregon Research Institute, Eugene, USA; 5 WHO collaborating Center for Research in Human Reproduction Unit of Epidemiology, China; 6 Hangzhou Center for Disease Control and Prevention, China

## Abstract

**Background:**

The Chinese National People's Congress ratified the WHO Framework Convention on Tobacco Control (FCTC) on 27 August 2005, signaling China's commitment to implement tobacco control policies and legislation consistent with the treaty. This study was designed to examine attitudes towards four WHO FCTC measures among Chinese urban residents.

**Methods:**

In a cross-sectional design study, survey data were collected from two Chinese urban cities involving a sample of 3,003 residents aged 15 years or older. Through a face-to-face interview, respondents were asked about attitudes toward four tobacco control measures developed by the WHO FCTC. Data on the four dependent measures were analyzed using multivariate logistic regression analyses. Using descriptive statistics, potential change in smoking behavior that smokers might make in response to increasing cigarette prices is also reported.

**Results:**

81.8% of the respondents in the study sample supported banning smoking in public places, 68.8% favored increasing the cigarette tax, 85.1% supported health warnings on cigarette packages, and 85.7% favored banning tobacco advertising. The likelihood to support these measures was associated with gender, educational level, and personal income. Smokers were less likely to support these measures than non-smokers, with decreased support expressed by daily smokers compared to occasional smokers, and heavy smokers compared to light smokers. The proportion of switching to cheaper cigarette brands, decreasing smoking, and quitting smoking altogether with increased cigarette prices were 29.1%, 30.90% and 40.0% for occasional smokers, respectively; and 30.8%, 32.7% and 36.5% for daily smokers, respectively.

**Conclusion:**

Results from this study indicate strong public support in key WHO FCTC measures and that increases in cigarette price may reduce tobacco consumption among Chinese urban residents. Findings from this study have implications with respect to policymaking and legislation for tobacco control in China.

## Background

As the world's largest producer and consumer of tobacco, China has a significant higher smoking prevalence than most other countries. For example, data from the 2002 national prevalence survey indicated an overall prevalence of 35.8% among China's population 15 years of age and above, with 66.0% ever smokers reported for males and 3.1% for females [1]. These numbers translate to approximately 350 million ever smokers in China. Epidemiological data suggests that patterns of tobacco use in China vary by occupational groups (71% among factory workers to 56% among teachers) and educational attainment (72% among those with no education to 54% among those with a college education) [1,2]. As a result, smoking-related diseases are becoming epidemic and of significant public health concern [3,4]. Studies of tobacco-related mortality in China have shown that tobacco smoking already accounts for approximately 800,000 deaths annually [5]. Studies have shown that cigarette smoking is the key behavioral risk factor in the rising incidence of cancer, cardiovascular disease, and Chronic Obstructive Pulmonary Disease (COPD) [6-8]. For example, the prevalence of COPD in China is estimated to exceed 3%, or 25 million people, of whom 72% were smokers [8]. Although China is in an early stage of a tobacco epidemic, it has suffered a tremendous burden from tobacco- induced diseases. Nearly 800,000 Chinese die each year as a result of tobacco use [4] and the number will increase to 2 million by the year 2025, if current smoking rates continue [9].

Because of China's large smoking population and high rates of mortality attributable to cigarette smoking, several researchers argued for more effective, broadly applied smoking control strategies in China which could prevent at least 50 million deaths [2,10]. In response to the global tobacco epidemic, in 1999, the WHO initiated the WHO Framework Convention on Tobacco Control (FCTC), which was subsequently endorsed unanimously by WHO member states on 21 May, 2003 to become a legally-binding international public health treaty. The WHO FCTC requires nations to implement a range of tobacco control policies. The Chinese National People's Congress ratified the WHO FCTC on 27 August 2005, signaling China's commitment to implement all the provisions specified under the WHO FCTC. While an important step toward tobacco control in China, the extent to which public support and public opinion on the FCTC measures remains largely unknown. From a public health perspective, such information may prove of great importance with respect to understanding the degree to which public support and opinions may have on the successful adoption of tobacco control policies and legislation in China. Therefore, this study was designed to gather baseline information about attitudes toward four key tobacco control measures developed by the FCTC: banning smoking in public places, raising taxes on tobacco products, banning tobacco advertising, and labeling cigarette packets with health warnings. In addition, the paper also examined the relationship between price increase for cigarettes and smokers' intention to behavioral change.

## Methods

### Study design

This study used a cross-sectional, multi-stage sampling design in which two urban cities were first pre-selected (Stage 1), followed by the selection of residential districts within each city (Stage 2), identification of blocks of apartment buildings within each district (Stage 3), and sampling households within each block of buildings (Stage 4). Details are presented below.

The study areas covered two urban cities in China: Xian and Hangzhou, which are the capital cities of Shaanxi and Zhejiang provinces, respectively, located in the northwest and southeast areas in China. Within each sampling frame, two residential districts with high density of family households were selected. From each of these residential districts, blocks of apartment buildings were randomly selected using "Jiedao" (a sub-district neighborhood administration), from which a city-identified list of family households was used to randomly sample households using a proportional sampling method. Within each household, individuals aged 15 years and over who lived in their residences for at least one year were identified. One respondent was selected from each family using a randomized method.

### Procedures

Once an individual was identified and agreed to participate, a face-to-face interview was scheduled. All interviews were conducted in a private setting by trained research interviewers who administered a brief questionnaire, which included information on demographics, smoking history, and attitudes. The same interview protocol was used across both cities to ensure identical interview and data collection procedures. The study was approved by the ethics committee at Medical Center, Zhejiang University and informed written consent was obtained from all study respondents before the study began.

### Measures

The survey questionnaire covered four categories: (a) demographics, (b) smoking status, (c) attitudes towards tobacco control, and (d) intention to change smoking.

#### Demographics

Information regarding study region (Xian, Hangzhou), age (< 20 years, 20–29 years, 30–39 years, 40–49 years, and 50 years and over), gender, education (Junior school or lower, high school, college or above), marital status (never, married, others), occupation (professionals, leaders, clerks, commerce and service, operation workers, never employed, unemployed, retired, others), and average annual income/person (< 10000 RMB, 10000–14999 RMB, 15000 RMB or greater) was obtained through self-report.

#### Smoking status

Information regarding current smoking, frequency, number smoked, and smoking history was assessed through self-report. A current smoker was defined as someone who smoked in the past month, daily smokers were defined as those who smoked every day, and occasional smokers were defined as those who smoked on some days within the past month, and a former smoker was those who smoked in everyday in the past (for at least 6 months) but were not smoking (not a single cigarette) for the preceding one month.

#### Attitudes towards tobacco control

Four WHO FCTC measures were used: smoking bans in public places, increasing cigarette taxes, tobacco advertising bans, and health warnings on cigarette packages. Respondents were asked to respond whether or not they favored each of these measures. Respondents were also asked two questions regarding the allocation of cigarette tax revenues: (1) Are you in favor of using tobacco tax revenues smoking control and health services? (2) Are you in favor of using the tobacco tax revenues for government expenses?

#### Intention to change smoking behaviour

This measure was designed to assess smoking respondent's intent to change smoking behavior with increasing cigarettes prices among daily smokers. Specifically, respondents were asked: (1) Would an increase in cigarettes price affect your smoking behavior? (2) If "yes", how would your smoking change? Respondents were given the choice of (a) Switch to cheaper tobacco brands; (b) Decrease smoking; or (c). Stop smoking completely. For each of these response choices, respondents were further asked: (1) What percentage of increase in the price of cigarettes might make you switch to cheaper tobacco brands? (2) What percentage of increase in the price of cigarettes would make you decrease smoking? (3) What percentage of increase in the price of cigarettes would make you stop smoking "c" completely?

To assess the reliability of responses, we telephone interviewed a randomly selected 10% of the total subjects (N:306). We re-assessed their responses on smoking status, attitudes towards four key FCTC measures and so on. Results indicated adequate temporal stability, with values ranging from 0.87 to 0.96 (mean value = 0.92), With respect to responses of test-retest about whether or not smoke is completely agreement, which indicating high agreement between the two modes of assessments.

### Data analyses

Chi-square tests were conducted to examine differences between the four WHO FCTC variables and demographic measures of the study samples. Subsequent multivariate logistic regression analyses were conducted to assess the association between attitudes and various demographic, smoking status, and intention measures. In these analyses, independent variables were region (cities(1 = Xian; 2 = Hangzhou), gender(1 = male; 2 = female) age, education, marital status, occupation, personal income, and smoking status(1 = non-smokers; 2 = occasional smokers; 3 = lighter daily smokers; 4 = heavy daily smokers). Dependent variables were the four tobacco control measures, operationalized as a binary variable with 1 (= in favor) and 2 (= not in favor). Regression analyses were ran on each of the four tobacco control dependent variables. Cumulative percent analyses were used to indicate cumulative percent of changing smoking behavior among daily smokers as cigarette prices increased. All analyses were carried out using SAS.

## Results

### Descriptive statistics of the sample

Of the total 3,564 households contacted, 3,210 were eligible. Of these, 153 refused to be interviewed, resulting in a response rate of 95% (3,057/3,210) from the target population. Of the total questionnaires collected, 54 questionnaires were incomplete due to missing of key information and, therefore, were excluded from analyses, leaving a sample size of 3,003 respondents for the final analyses. Table 1 shows the demographic characteristics of the survey sample. Both the number of respondents across the two sampled cities and gender distribution were almost equivalent, and the median age category was between 30 and 39 years. About 66% of the respondents had completed high school education. The majority reported married (70%) and employed (73%). In general, the sample characteristics are comparable to the two national surveys [11,12], with the exception that it exhibited a higher level of education compared to the national samples, possibly due to the fact that the current sample was drawn from two major metropolitan cities in China.

**Table 1:** Demographic characteristics of the sample, Urban Chinese Residents

group	n	%	X1*	X2**
Region				
Hangzhou	1443	48.1	-	-
Xian	1560	51.9	-	-
Gender				
Male	1575	52.5	49.9	52.2
Female	1427	47.5	50.1	47.8
Age				
<20 years	266	8.9	5.1	3.8
20–29	525	17.5	14.9	15.8
30–39	639	21.3	20.7	29.4
40–49	875	29.1	29.7.	33.3
50+ years	698	23.2	29.6	17.6
Education				
Junior school and below	716	8.9	5.1	3.8
High school	1271	42.3	25.4	44.6
Technological college	599	19.9	-	18.6
College and above	417	13.9	9.1	11.9
Marital status				
Never been married	531	17.7	19.1	13.6
Married	2095	69.8	74.3	83.9
Others(divorced, widowed and so on)	377	12.5	2.9	2.5
Occupation				
Leader^◆^	247	8.2		8.5
Clerks	432	14.4		21.0
Professional	278	9.3		9.7
Commerce and service	587	19.5		17.4
Operation workers	313	10.4		5.6
Never been employed	188	6.3		3.4
Unemployed	208	7.3		10.1
Retired	412	13.7		8.8
Others^◆◆^	328	10.9		15.4
				
Average Annual Income/person (RMB)				
				
<10000yuan	991	33.0		
10000-yuan	833	27.7		
15000-yuan	1179	39.3		

*China population statistics yearbook data [11]; ** National urban residents stress survey data [12].

^◆^Leader of governmental or party agencies, enterprises or institutions; ^◆◆^Others included students and army.

### Smoking rates

Overall, the smoking prevalence for the study sample was 41.4% (n = 1,243). Of which, 35.2% were identified as daily smokers (n = 1,057), and 6.2% (n = 186) were occasional smokers. A significantly higher proportion of male daily smokers (64.3% versus 2.20% (female); χ^2 ^= 1263.38, P < 0.01), and occasional smokers (11.0% (male) versus 0.7%(female) ; χ^2 ^= 138.17, P < 0.01). Across the two cites, a significantly higher proportion of daily smokers was identified in Xian, compared to those in Hangzhou (40.8% versus 29.0%; χ^2 ^= 45.75, P < 0.01); However, there was no statistical significant difference between occasional smokers across the two cities (6.7% versus 5.8%; χ^2 ^= 1.07, P > 0.05.); and daily smoking prevalent was significantly higher in Xian than in Hangzhou (70.4% versus 56.6%; χ^2 ^= 32.55, P < 0.01) for male, but this difference did not show (2.3% versus 2.8%; χ^2 ^= 1.07, P > 0.05.) for female. The former smoking rate was 12.3%. The average number of daily cigarettes smoked by daily smokers was 13.94 (SD: 9.58).

### Attitude towards key tobacco control measures from the WHO FCTC

Overall, there was evidence for supporting the four FCTC measures. Approximate eighty-two percent (95% C.I: 81.1%–82.5)of the respondents reported being in favor of a ban on smoking in public places, 68.8% (95% C.I: 68.0%–0.70.0%) support increasing the cigarette tax, 85.1% (95% C.I: 84.5%–85.7%) favor health warnings, and 85.9% (95% C.I: 85.1%–86.3%) support a ban on tobacco advertising. Table 2 displays results from multiple regression analyses.

**Table 2:** Logistic regression model to predict favorable attitude towards the four FCTC measures (N = 3,003)

Group	N	Banning smoking in public places X (in favor)% OR(95%C.I)			Increasing cigarette taxes X(in favor)% OR(95%C.I)			Health warnings X(in favor)% OR(95%C.I)			Banning tobacco advertising X(in favor)% OR(95%C.I)		
Region													
Hangzhou	1443	1337	92.7	1.00	1067	74.6	1.00	1327	92.0	1.00	1201	83.2	1.00
Xian	1560	1120	71.8	0.20(0.17,0.28)**	990	63.5	0.66(0.55,0.78)**	1230	48.9	0.35(0.28,0.45)**	1378	88.4	1.53(1.22,1.90)**

Gender													
Male	1595	1155	72.4	1.00	951	59.6	1.00	1263	79.2	1.00	1344	84.3	1.00
Female	1408	1302	92.5	1.61(1.12, 2.32)**	1115	79.2	1.38(1.06,1.79)*	1294	91.9	0.86(0.57,1.28)	1235	87.1	0.79(0.56,1.13)

Age													
< 20 y	266	223	85.0	1.00	197	74.1	1.00	226	85.0	1.00	223	83.8	1.00
20-	525	438	83.4	1.38(0.87,2.19)	378	72.0	0.96(0.67,1.34)	464	88.4	1.77(1.12,2.78)	464	88.4	1.18(0.76,1.85)
30-	639	564	88.3	1.24(0.77,2.00)	426	66.7	0.58(0.40,0.84)**	558	87.3	0.78(0.49,1.25)	512	80.1	0.65(0.41,1.01)
40-	875	710	81.1	0.97(0.62,1.52)	598	68.3	0.81(0.56,1.15)	745	85.5	0.85(0.55,1.33)	773	88.4	1.05(0.67,1.36)
50-	698	519	74.4	0.50(0.53,0.84)**	467	66.9	0.67(0.47, 0.95)*	564	80.8	0.65(0.43,0.99)*	608	87.0	1.13(0.75,1.70)

Education													
Junior school and low	716	594	83.0	1.00	466	65.1	1.00	605	84.5	1.00	414	85.8	1.00
High School	1271	1006	79.2	0.80(0.61,1.06)	846	66.6	1.14(0.92,1.43)	1050	82.6	0.84(0.63,1.13)	1072	84.1	0.99(0.76,1.31)
College and above	1016	857	84.4	0.92(0.65,1.31)	754	74.5	1.32(1.01,1.72)*	902	88.8	1.46(1.02,2.08)*	893	87.9	1.38(1.00,1.92)*

Marital status													
Never	531	430	81.0	1.00	386	72.7	--	446	84.1	1.00	449	84.6	1.00
Married	2095	1752	83.6	1.62(1.13,2.30)**	1459	69.6	1.17(0.88,1.72)	1828	87.3	1.94(1.38,2.73)**	1812	86.5	1.51(1.05,2.17)
Others(Divorced, widowed and so on)	377	275	72.9	0.80 (0.52,1.23)	221	58.6	0.62(0.44,0.88)**	283	75.1	0.76(0.52,1.11)	318	84.4	0.90(0.59,1.39)

Occupation													
Professional	278	234	84.2	1.00	212	76.3	1.00	241	86.7	1.00	249	90.0	1.00
Leader◆	247	198	80.2	0.67(0.40,1.11)	176	71.3	0.82(0.54,1.24)	208	84.1	0.74(0.44,1.27)	205	83.0	0.58(0.35,0.98)*
Clerks	432	382	88.4	0.92(0.57,1.50)	318	73.6	0.79(0.54,1.15)	385	89.1	0.93(0.57,1.54)	352	81.5	0.57(0.36,0.91)*
Commerce and service	587	504	85.7	0.81(0.52,1.26)	394	67.1	0.60(0.42,0.86)**	516	87.9	1.01(0.62,1.62)	508	86.5	0.86(0.54,1.38)
Operation workers	313	221	70.6	0.58(0.37,0.91)*	192	61.3	0.70(0.47,1.05)	244	78.0	0.82(0.50,1.25)	256	81.8	0.59(0.35,0.98)*
Never been employed	188	146	77.7	0.55(0.33,0.93)*	116	61.7	0.58(0.37,0.91)*	162	86.2	1.17(0.64,2.14)	164	87.2	0.85(0.47,1.56)
Unemployed	208	173	79.4	0.61(0.36,1.04)	119	54.6	0.38(0.25,0.59)**	177	81.2	0.62(0.36,1.09)	199	91.3	1.27(0.67,2.38)
Retired	412	315	76.5	0.63(0.39,1.00)	284	68.9	0.83(0.55,1.25)	342	83.0	1.08(0.64,1.82)	375	91.0	1.21(0.70,2.11)

Others◆ ◆	328	284	86.6	0.72(0.42,1.21)	255	77.7	0.85(0.55,1.31)	282	86.0	0.80(0.44,1.47)	271	82.6	0.52(0.32,0.86)*

Average Annual Income/person(RMB)													
< 10000	991	839	84.7	1.00	644	65.0	1.00	848	85.6	1.00	847	85.5	1.00
10000-	833	629	75.7	0.73(0.53,1.02)	564	67.7	1.25(1.01,1.55)*	690	82.8	0.91(0.69,1.21)	715	86.0	1.07(0.82,1.41)
15000-	1179	989	83.9	0.96(0.66,1,41)	858	72.8	1.27(1.03,1.57)*	1019	86.4	0.80(0.60,1.07)	1017	86.3	1.27(0.96,1.66)
Smoking status													
Non-smoking	1760	1619	92.0	1.00	1388	78.9	1.00	1630	92.6	1.00	1558	88.5	1.00

Occasional smoking	187	158	84.5	0.60(0.36,1.01)	106	56.7	0.42(0.29,0.61)**	147	78.6	0.28(0.18,0.42)**	140	74.9	0.39(0.27,0.56)**
Daily smoking													
Light(< = 10 cigarettes/day)	531	360	67.8	0.23 (0.16,0.34)**	320	60.3	0.50(0.37,0.67)**	418	78.2	0.29(0.18,0.42)**	434	81.7	0.58(0.44,0.76)**
Heavy(> 10 cigarettes/day)	525	320	61.0	0.21(0.14,0.30)**	252	48.0	0.31(0.23,0.41)**	362	69.0	0.17(0.13,0.22)**	447	85.3	0.73(0.55,0.98)*

◆ Leaders of governmental or party agencies, enterprises or institutions; ◆◆ Others included students and army and other uncategorized OR(odds ratio) is a way of comparing whether the probability of favorable attitude towards the four FCTC measure is the same for two groups,*p < 0.05, **p < 0.01

Significant predictors to support bans on smoking in public places were: being residents of Hangzhou (Odds ratio (OR):4.35), females (OR:1.61), being younger than aged 50 years or above (OR:2.00), being married (OR:1.62), being Professional (OR:1.49), never been employed (OR:1.82); and being non-smokers (light daily smokers (OR:0.23), and heavy daily smokers (OR:0.21).

Significant predictors to support increasing tobacco taxes were: being residents of Hangzhou (OR:1.28); females (OR:1.38); being younger than aged 30 years (OR:1.72) and 50 years or above (OR:1.49); attained college degree education or above (OR:1.32); never been married (R:1.67); being Professional (OR:1.67); never been employed (OR:1.72) or Unemployed (OR:2.63); having an average personal annual Income of 1,000 yuan (OR:1.25) or 15,000 yuan or above (OR:1.27); and being never smoker (occasional smokers, OR:0.42; light daily smokers, OR:0.50; heavy daily smokers OR:0.31).

Significant predictors to support health warnings on cigarette packages were: being residents of Hangzhou (OR:2.54); being younger than 50 years or above (OR:1.54); attaining college degree education or above (OR:1.46); being married (OR:1.94); and being non-smokers (occasional smokers, OR:0.28; light Daily smokers, OR:0.29, Heavy Daily smokers OR:0.17).

Significant Predictors to support bans on tobacco advertising were: being residents of Hangzhou (OR:0.65); attaining education to College and above (OR:1.38); being Professional (OR:1.72) or Clerks (OR:1.75) or others (OR:1.92); being non-smokers (occasional smokers, OR:0.39, light daily smokers, OR:0.58, heavy daily smokers OR:0.73).

We also examined possible interaction between smoking status and city of residence regarding increasing in cigarette tax. Analyses showed a significant smoking status by city of residence interaction effect: OR = 3.52 (95% CI: 1.67,7.45) for occasional smokers; OR = 2.48 (95% CI: 1.38,4.47) for light smokers; and OR = 1.87 (95% CI: 1.02,3.42) for heavy smokers, which meant the OR values were lower in Hangzhou than Xian and indicated that smokers in Hangzhou expressed less support to increasing cigarette tax compared to non-smokers across all levels of smokers. However, further analyses showed a significant difference between Hangzhou and Xi'an in relation to non-smokers (84.5% versus 72.4%; χ^2 ^= 38.31, P < 0.01), and no significant differences were observed in other categories of smoking status across the two cites. The above results suggested that non-smoking residents in Hangzhou expressed more support for cigarette tax increases.

We would like to clarify that there were no interaction effects observed in other three tobacco control measures in the study.

Finally, respondents tended to support raising tobacco taxes if the revenues were channeled for smoking control and health services (74.2%; 95% C.I: 71.6%–0.75.0%). The likelihood to support tobacco tax increases was lower (48.1% 95%C.I:47.2%–49.0%) if the revenues were intended for government use.

### Cigarette price increasing and behavioral change

The proportion of respondents who intended to change behavior differed between current smokers (25.6%), occasional smokers (29.4%), and daily smokers (24.9%) (see Table 3). There were no significant differences between daily smokers and occasional smokers (χ^2 ^= 0.739, P = 0.691). The patterns of change (i.e. switching to cheaper brands, decreasing consumption and quitting) reported by smokers were almost similar between different categories of smokers (see Table 4). There were no significant differences between daily smokers and occasional smokers (χ^2 ^= 0.239, P = 0.888).

**Table 3:** Cigarette price increasing and behavioral change intention

Group	N	Change		No change		Uncertainty	
		
		n	% (95%C.I)	n	% (95%C.I)	n	% (95%C.I)

Current smokers	1243	318	25.6(24.3,26.8)	406	32.7(31.3,34.0)	519	41.8(39.8,42.6))
Occasional smokers	187	55	29.4(26.1,32.7)	47	25.1(22.0,28.3)	85	45.5(41.8,49.1)
Daily smokers	1056	263	24.9(23.6,26.2)	359	34.0(32.5,35.5)	434	41.1(39.6,42.6)

**Table 4:** Type of behavioral change with Cigarette price increasing

Group	N	Switch to cheaper brands		Decreasing consumption		Quitting	
		
		n	% (95%C.I)	n	% (95%C.I)	n	% (95%C.I)

Current smokers	318	97	30.5(22.9,35.2)	103	32.4(30.836.1)	118	37.1(35.6,41.1)
Occasional smokers	55	16	29.1(14.7,25.5)	17	30.9(24.7,37.1)	22	40.0(33.4,46.6)
Daily smokers	263	81	30.8(28.0,33.7)	86	32.7(29.8,35.6)	96	36.5(33.5,39.5)

Table 5 shows the relationship between cigarette price increases and smoking behavioral changes for daily smokers. The entries show that reported intention to change smoking behavior increases with increasing cigarettes prices. Smokers were more likely to report switching to cheaper brands followed by a reduction in smoking; quitting smoking was the least likely to be reported as an intended change. For example, a projected 10% increase in the price of cigarettes would make 16.3% of smokers to reduce their smoking consumption and 2.1% of smokers to consider quitting.

**Table 5:** Cumulative percent of behavioral change with increasing cigarettes price for daily smokers. **

Switch to cheaper brands			Reducing consumption			Considering quitting		
Price increase(%)	Overall(%)	Cumulative (%)	Price increase(%)	Overall (%)	Cumulative (%)	Price increase(%)	Overall (%)	Cumulative (%)

1	2.5	2.5	5	1.2	1.2	5	1.1	1.1
2	1.2	3.7	7	1.2	2.4	10	1.1	2.2
5	9.9	13.6	10	14.0	16.4	20	3.1	5.3
8	1.2	14.8	15	1.2	17.6	30	4.2	9.5
10	16.1	30.9	20	17.4	35.0	50	21.9	31.4
13	1.2	32.1	30	13.9	48.9	80	6.2	37.6
15	2.5	34.6	35	2.3	51.2	100	22.9	60.5
20	18.5	53.1	40	3.5	54.7	104	1.1	61.6
23	1.2	54.3	50	12.8	67.5	120	6.2	67.8
24	1.32	55.5	54	4.7	72.2	135	3.1	70.9
30	11.1	66.6	56	1.2	73.4	139	3.1	74.0
32	2.5	69.1	57	1.2	74.6	150	1.1	75.1
35	2.5	71.6	58	2.3	76.9	200	8.3	83.4
40	3.7	75.3	59	1.2	78.1	300	5.2	88.6
45	1.2	76.5	60	2.3	80.4	400	5.2	93.8
48	2.5	79.0	66	1.2	81.6	500	3.1	96.9
50	13.6	92.6	70	2.3	83.9	600	1.1	98.0
53	1.2	93.8	78	1.2	85.1	740	1.1	99.1
76	1.2	95.0	80	5.8	90.9	750	1.1	100.0
100	3.7	98.7	100	7.0	97.9			
300	1.2	100.0	200	1.2	99.1			
			500	1.2	100.0			

**The price increase (%) is projected based on current prices.

## Discussion

This survey data provide empirical evidence that urban residents are strongly supportive of the key WHO FCTC measures in two major metropolitan cities in China. The rate of support ranged from as high as 85.7% to as low as 68.8%. Respondents in this study had higher (85.7%) support for tobacco advertising bans. Although underlying reasons for the high level of support are unclear, one speculation is that it may be due to respondents' dissatisfaction to the advertisement. Similarly, the majority of the respondents in the survey reported exposure to tobacco advertisements (78%), mainly from television and public boards. The proportion, however, is lower compared to those reported in Massachusetts, USA (88.0%)[13]. This finding suggests that strict law enforcement would be accepted by most smokers in China. Also, the high support for health warnings in this study reflects the information gap for health related problems among the smokers. Lastly, about 82% of the respondents favored ban on smoking in public places, which is similar to the findings among Australian restaurant workers (81%) [14], but lower than that reported by the general public in Taiwan (91.4%)[15]. The more favorable support in Taiwan might be due to the extensive Governmental support to combat tobacco use in Taiwan which was supplemented by counter advertisement and awareness campaign [16]. Taken together, the different demographic predictors that we have identified for four key measures indicate that interventions need to be customized to reach the public for each of the tobacco control measures.

Although some studies reported that smokers are less likely to support a cigarette tax than non-smokers [17], about two-third (68.8%) of our respondents was supportive. It is higher than that reported among the general public in Taiwan [17]. Consistent with the previous findings [18], our study revealed that smoking behavior played a role in their support for key tobacco control measures. In this study, support was low among daily smokers compared to occasional smokers, and heavy smokers compared to light smokers. This reflects the fact that self-interest plays an important role in influencing social attitudes toward smoking control policies in China.

Results of this study not only shed important light upon the significance of socio-economic characteristics in determining the attitude toward tobacco control measures but also are consistent with those reported by others [15,19]. For example, Tsai et al showed that the reported rates of potentially changing smoking behavior in response to the earmarked tax were quite different between smokers who were better-off financially than those who were less well-off:17.7% versus 24.4% (P < 0.05) [15]. The support for tobacco excise taxes was stronger among younger persons, females, those with higher education, racial/ethnic minorities, and smokers with children [20]. Similarly, Brownson et al reported that better educated people, white-collar workers, and nonsmokers were more likely to support bans than those who were less educated, blue-collar workers, and smokers[18].

We found that there are marked differences in support for policies between the two cities. Smoking prevalence was also different in the two cities. For example, daily and occasional smoking rate in Xian was 40.8% and 6.7%; the corresponding figure in Hangzhou was 29.0% and 5.8%. It reflects the fact that there are significant social and economic differences between the two regions. It should be noted that economically Xian is less developed than Hangzhou. It suggests that people's lifestyle and beliefs may be influenced by the social and economic developments in the area. These changes may have facilitated social and environmental change with respect to people's health awareness and health belief.

Consistent with the literature [13,19-21], in this study, there was a strong association between cigarette price increasing and intentions to change smoking behavior. The most commonly reported reaction of smokers in the state of Massachusetts to cigarette price increases of 15% in 1993 was to consider quitting (35.0%), followed by changing to a cheaper brand (28.4%), then to reducing the number of cigarettes smoked (17.0%) and quitting (3.7%) [3]. In Taiwan, 27.2% of smokers changed to a cheaper brand, 25.4% of smokers reduced the number of cigarettes smoked after the price rose NT$5(14.2%) from 2001–2002 [15]. We found that the proportion of switching to use cheaper brands is higher and the proportion of reducing cigarette consumption and considering quitting are lower in Chinese mainland than elsewhere. That may be explained by the difference of health belief as influenced by the level of social and economic development. In this study our subjects came from Hangzhou and Xian, the economic development level of the former (annual revenue/person: 19027RMB (in 2006) [22] than the later (annual revenue/person: 10074RMB (in 2006) [23] We also found a difference between the two cities, which supports that socio-economic and environmental factors play a role in intention to change behavior [24].

The results from the interactive model testing indicate that non-smoking residents in Hangzhou expressed more support for cigarette tax increases than those in Xian, which may be explained by the fact that, compared to the city of Xian, the city of Hangzhou is more socially and economically developed which may help raise better health awareness and belief s among its residents.

In this study, the response to price increases is not linear. Rather, it appears to demonstrate a threshold effect, with initial slow rise in intention to change at lower levels of price increases, followed by rapidly increasing reported intention to change, eventually reaching a saturation period. This supports the general consumer behavior response rules for price increasing, which consumers' response to price increasing for different items show less sensitive period, sensitive period, and saturation period [25]. Supporting the report of Chaloupka et al [26], our findings also suggest that there is a clear relationship between increase tobacco taxes and reduced tobacco consumption[26].

There are few limitations of this study. First, this study was cross-sectional, with no causality implied. Second, the study sample frame included urban environments involving two cities with sufficient variability in socio-economic conditions. As such, the findings may not be generalized to all metropolitan areas of the nation. Similarly, because of the urban-focused study areas, the findings can not be extrapolated to rural populations. Third, the study assessed behavioral intentions, which is different from actual behavior change. Respondents who reported an intention might not actually change behavior in practice. Future studies should measure actual behavior change while assessing impact of price increase on behavior change or while assessing the impact of FCTC measures.

## Conclusion

This survey study shows general support of tobacco control measures among Chinese urban residents and that increases in cigarette price are likely to be associated with smoking behaviors. These findings provide policy makers with a consensus of public opinion on key WHO FCTC measures and their association with major demographic characteristics of urban residents in China. Such information is of great policy-making importance and public health relevance for tobacco control initiatives that are urgently needed to decrease the current smoking prevalence in China.

## Competing interests

The author(s) declare that they have no competing interests.

## Authors' contributions

TY conceived of the study, conceptualized ideas, supervised its conduct and data analyses, DD and HX conducted the data collection, Abu Abdullah and FL provided technical support on the data analysis and finalizing the draft manuscript and WY, JW helped to interpret findings and contributed to the text. All authors reviewed drafts of the manuscript and approved the version to be published.

## Pre-publication history

The pre-publication history for this paper can be accessed here:


